# Synthesis of Mono-Amino Substituted γ-CD: Host–Guest Complexation and In Vitro Cytotoxicity Investigation

**DOI:** 10.3390/molecules27051683

**Published:** 2022-03-04

**Authors:** Fadwa Odeh, Fedaa Adaileh, Walhan Alshaer, Hamdi Nsairat, Dana A. Alqudah, Areej M. Jaber, Abeer Al Bawab

**Affiliations:** 1 Department of Chemistry, School of Science, The University of Jordan, Amman 11942, Jordan; fedaa.adaileh@gmail.com (F.A.); drabeer@ju.edu.jo (A.A.B.); 2 Cell Therapy Center, The University of Jordan, Amman 11942, Jordan; pharmd.dana.alqudah@gmail.com; 3 Pharmacological and Diagnostic Research Centre, Faculty of Pharmacy, Al-Ahliyya Amman University, Amman 19328, Jordan; hammdi2000@yahoo.com (H.N.); a.jaber@ammanu.edu.jo (A.M.J.); 4 Hamdi Mango Center for Scientific Research, The University of Jordan, Amman 11942, Jordan

**Keywords:** cyclodextrins, γ-CDs, host–guest complexes, curcumin, mono-amino substitution, weak base modification, drug carrier

## Abstract

Cyclodextrins (CDs) are cyclic oligosaccharides which can trap hydrophobic molecules and improve their chemical, physical, and biological properties. γ-CD showed the highest aqueous solubility with the largest cavity diameter among other CD types. The current study describes a direct and easy method for nucleophilic mono-aminos to be substituted with γ-CD and tested for their ability to host the guest curcumin (CUR) as a hydrophobic drug model. The mass spectrometry and NMR analyses showed the successful synthesis of three amino-modified γ-CDs: mono-6-amino-6-deoxy-cyclodextrine (γ-CD-NH_2_), mono-6-deoxy-6-ethanolamine-γ-cyclodextrine (γ-CD-NHCH_2_CH_2_OH), and mono-6-deoxy-6-aminoethylamino)-γ-cyclodextrin (γ-CD-NHCH_2_CH_2_NH_2_). These three amino-modified γ-CDs were proven to be able to host CUR as native γ-CDs with formation constants equal to 6.70 ± 1.02, 5.85 ± 0.80, and 8.98 ± 0.90 mM^−1^, respectively. Moreover, these amino-modified γ-CDs showed no significant toxicity against human dermal fibroblast cells. In conclusion, the current work describes a mono-substitution of amino-modified γ-CDs that can still host guests and showed low toxicity in human dermal fibroblasts cells. Therefore, the amino-modified γ-CDs can be used as a carrier host and be conjugated with a wide range of molecules for different biomedical applications, especially for active loading methods.

## 1. Introduction

Cyclodextrins (CDs) are cyclic oligomers of glucose units, derived from the bacterial enzymatic reactions of starch, consisting of six, seven, and eight D-glucopyranose units linked by α-1,4-glycosidic linkages, called α-, β-, and γ-cyclodextrin, respectively [1,2]. CDs have truncated cone shapes of different sizes, with a hydrophobic cavity resulting from the methylene and oxygen groups, and a hydrophilic exterior (upper and lower rim surface), due to the presence of primary and secondary hydroxyl groups. CDs are approved for use as food additives by the US Food and Drug Administration (FDA) and the World Health Organization–Food and Agriculture Organization of the United Nations (WHO–FAO) [3].

These compounds are widely used in the pharmaceutical field to improve the aqueous solubility, dissolution rate, chemical stability, and bioavailability of hydrophobic drugs [2]. In particular, the hydrophobic cavity of CDs enables them to encapsulate hydrophobic molecules of suitable sizes and generate inclusion complexes [4], which is called host–guest complexation [5]. This remarkable property of encapsulation of guest molecules is applied in many fields, including medicine, materials science, food, and agricultural industries [6,7,8,9]. The natural β-CD is the most commonly used CD in industrial products. β-CD accounts for about 70% of the global CD production, while α-CD is about 15%, and γ-CD is about 5% [10].

Recently, CDs have been utilized in cancer treatment due to their ability to enhance the aqueous solubility of hydrophobic anti-cancer flavonoids, such as curcumin and quercetin [11,12,13]. Compared with free curcumin, β-CD-curcumin has an enhanced solubility with a greater cellular uptake and a longer half-life, along with higher anti-proliferative and anti-inflammatory activities against leukemic, pancreatic, and tubulin surface cancer cells [11,12]. Yadav et al. reported that the CUR-β-CD complexation enhanced CUR solubility, and increased the CUR potency and the cellular uptake that induced apoptosis in tumor cells [11]. Moreover, the inclusion complex of the novel curcumin analogue CDF and β-cyclodextrin (1:2) enhanced the in vivo anti-cancer activity against pancreatic cancer [12]. Furthermore, Zhang et al. investigated the use of CUR-cyclodextrin complexes (CD15) as an approach to cancer chemoprevention. CD15 enhanced CUR delivery and improved its therapeutic efficacy compared to free CUR, both in vivo and in vitro [13]. Similarly, the aqueous solubility of quercetin improved linearly with increasing concentrations of three different β-CD derivatives: hydroxypropyl-β-CD, sulfobutyl ether-β-CD, and methylated-β-CD [14].

**Scheme 1:**
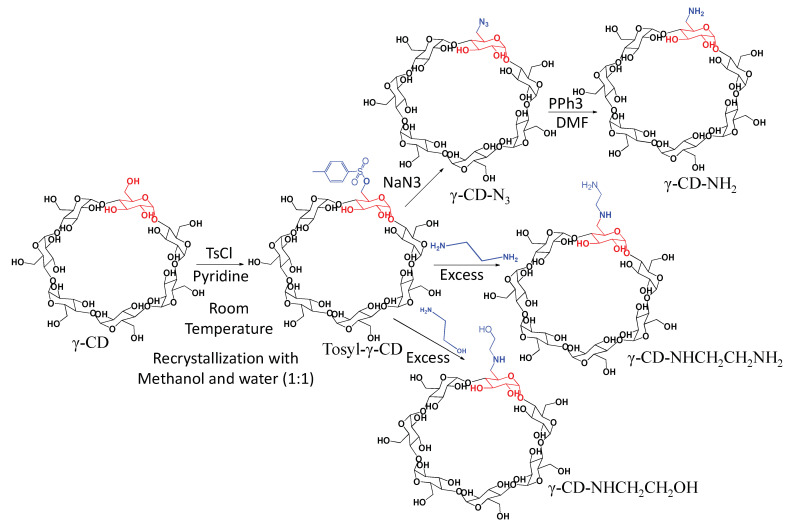
Schematic synthesis of modified γ-cyclodextrins.

The primary hydroxyl groups on the CDs’ exterior are the less hindered and more nucleophilic, which can be easily derivatized; therefore, a large variety of charged or uncharged derivatives is available [15,16]. Such modifications offer the possibility of enhancing the CDs’ physiochemical characteristics and opening the door for more pharmaceutical applications. These new CDs derivatives not only retain the binding ability of guest hydrophobic small and large molecules into the hydrophobic cavity, but also introduce new interactions with substituents at the rims, which are relatively well-hydrated [17]. Currently, the most commonly applied cyclodextrin derivatives are randomly methylated β-CD, 2-hydroxypropylated-β-CD, sulfobutylether-β-CD, sulfated-β-CD, and carboxymethyl-β-CD [6,17]. Commercially available modified CDs are generally limited to β-CD and to a multi- or random substitution of γ-CD [6]. Random substitution of CDs is not desirable for the hosts when binding selectivity is desired. Moreover, the resulting derivatives should be water-soluble and should not aggregate in the solution, so that accurate host–guest binding measurements can be carried out. γ-CD has the highest water solubility among other CDs, with a solubility of 23.2 g/mL, compared to 1.85 and 14.5 g/100 for γ-CD, β-CD, and α-CD, respectively [5]. Moreover, γ-CD has a larger hydrophobic cavity compared to other types of CDs, capable of accommodating large structure phytochemicals with potential anti-cancer effects [3]. γ-CD is considered safe due to its lower toxicity, and is the only native CD that undergoes complete digestion in the gastrointestinal tract [10]. However, the only commercially available derivatives for γ-CD are multi- and randomly substituted ones [18]. Accordingly, this work aims to provide a simple and easy synthesis protocol for water-soluble γ-CD derivatives that are singly substituted and still able to host a guest molecule (Scheme 1). Providing such a weak base and/or nucleophilic mono-amino substituted for γ-CD enables extra bioconjugation for a wide variety of small and large molecules, or even certain carboxylated polymers [19]. In addition, this weak base modification of CDs enhances the encapsulation efficiency of hydrophobic drugs in the liposomes aqueous core [15,20].

## 2. Results and Discussion

### 2.1. Characterization of Modified γ-CDs

#### 2.1.1. NMR and HR Mass Spectrometry

**Figure 1:**
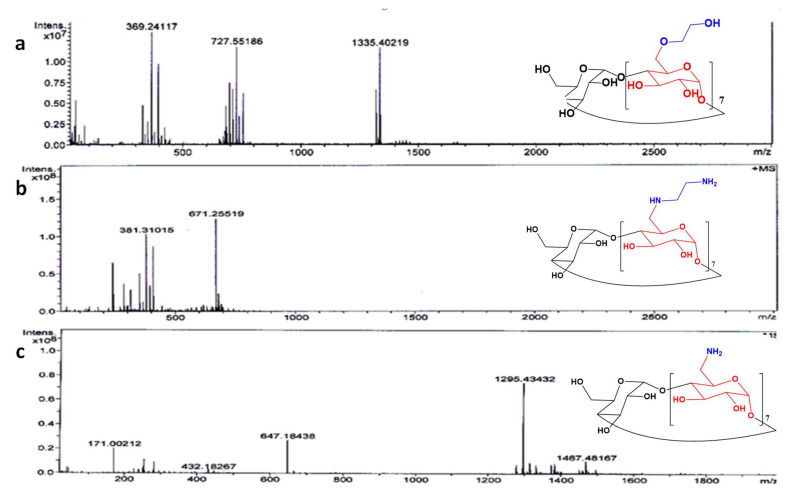
Mass spectra of (**a**) γ-CD-NHCH_2_CH_2_OH, (**b**) γ-CD-NHCH_2_CH_2_NH_2_, and (**c**) γ-CD-NH_2_ on the positive mode.

The formation of the three modified γ-CDs (γ-CD-NHCH_2_CH_2_OH, γ-CD-NHCH_2_CH_2_NH_2_, and γ-CD-NH_2_) was investigated using NMR and HR mass spectrometry. The HRMS (ESI) positive mode ([M + H]^+^) showed calculated and found mass values for γ-CD-NHCH_2_CH_2_OH (C_50_H_86_NO_40_) of 1340 and 1335.40, respectively (Figure 1a). The mono-substituted γ-CD-NHCH_2_CH_2_NH_2_ formation (C_50_H_86_N_2_O_39_) was also confirmed by the HRMS (ESI) positive mode ([M + H]^+2^) with calculated mass values of 671.1565 and 671.255, respectively (Figure 1b). Furthermore, the mono substitution for the formation of γ-CD-NH_2_ (C_48_H_81_NO_39_) was also confirmed with calculated mass values on the positive mode ([M + H]^+^) of 1295.4332 and 1295.4343, respectively (Figure 1c) [21].

**Figure 2:**
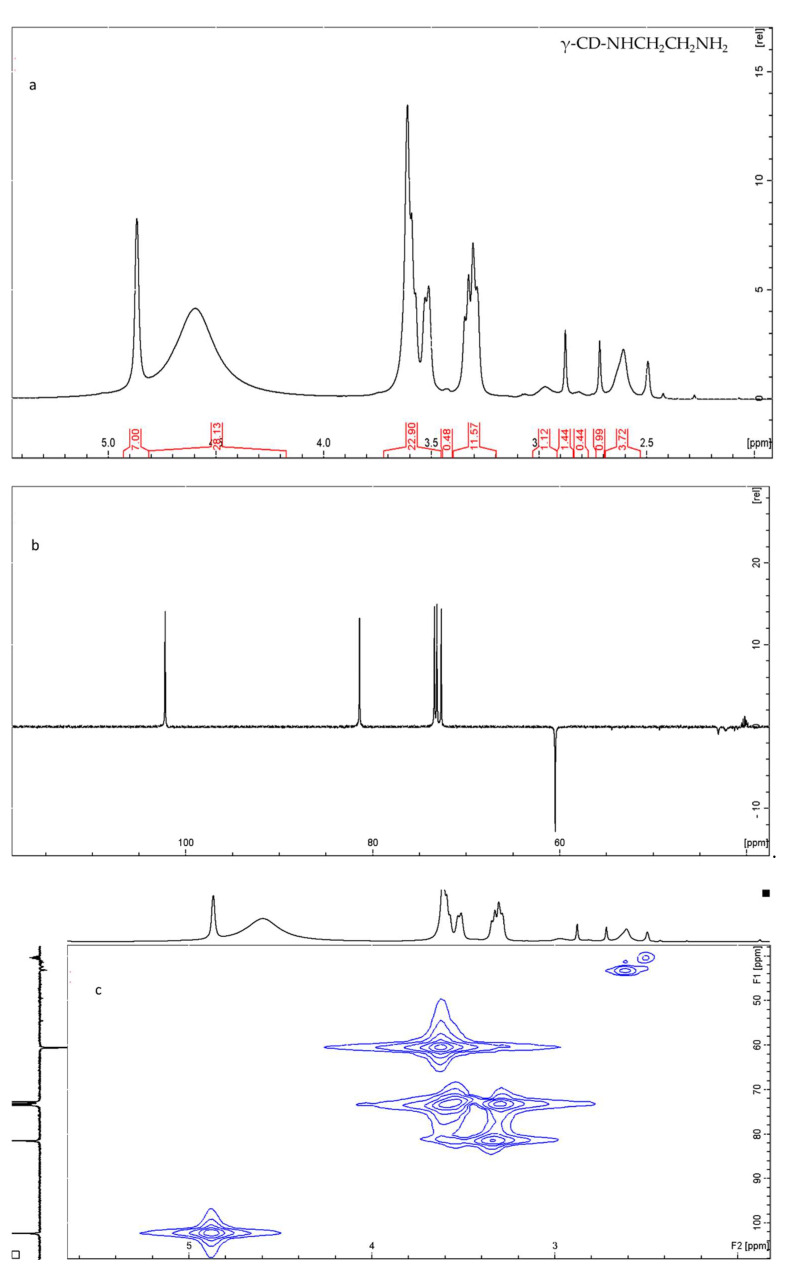
(**a**) ^1^H-NMR spectrum, (**b**) ^13^C-dept 135-NMR spectrum, and (**c**) ^1^H-^13^C-HMQC-NMR for γ-CD-NHCH_2_CH_2_NH_2_ in 80% *d*_6_-DMSO and 20% D_2_O at 25 °C.

The formation of the three modified γ-CDs (γ-CD-NHCH_2_CH_2_OH, γ-CD-NHCH_2_CH_2_NH_2,_ and γ-CD-NH_2_) was also confirmed using NMR spectroscopy (all spectra are in the Supplementary Information). The appearance of several new signals (compared with NMR data for γ-CD (Figure S1)) confirms the formation of these derivatives. For γ-CD-NHCH_2_CH_2_NH_2,_ the appearance of new signals at 2.4 ppm corresponds to the –CH_2_CH_2_- of the ethylene diamine group and have an integration ratio of ~4 to 7, relative to the C1H signal at 4.9 ppm (Figure 2a). In addition, several signals correspond to the NH, NH_2_, and C1–C6 protons of the glucose unit that underwent substitution. The ^13^C-dept 135 NMR spectrum for this compound is shown in Figure 2b. The most important signal is the CH_2_ signal at 43 ppm which appeared due to substitution. The assignment was further confirmed using ^1^H-^15^N-HMBC (Figure S2), showing the presence of two ^15^N environments. The mono-substituted γ-CD-NHCH_2_CH_2_OH was also confirmed using ^1^H-NMR and ^13^C-NMR (Figures S5 and S6). The appearance of two new signals at 2.1 ppm and 3.6 ppm indicates the formation of the derivative and the supporting mass spectrum (Figure 1a). In the ^13^C-NMR spectrum, two new signals appeared at 39.4 ppm and 31.1 ppm (Figure S6) for two CH_2_ carbon atoms.

#### 2.1.2. Thermo-Gravimetric Analysis (TGA)

**Figure 3:**
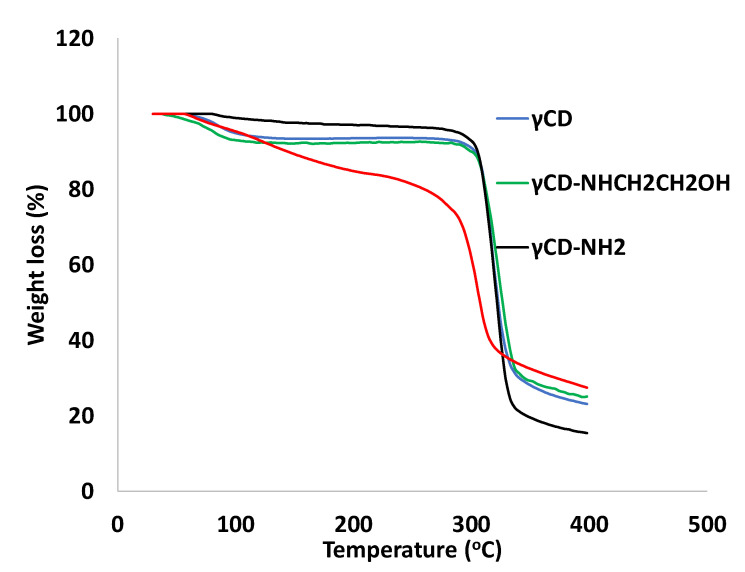
TGA thermograms of γ-CD-NHCH_2_CH_2_OH, γ-CD, γ-CD-NH_2_, and γ-CD-NHCH_2_CH_2_NH_2_.

The thermal profiles of γ-CD, γ-CD-NHCH_2_CH_2_NH_2_, γ-CD-NHCH_2_CH_2_OH, and γ-CD-NH_2_ are shown in Figure 3, with the temperature ranging between 30 °C and 400 °C. The thermal change occurred between 30 °C and 100 °C in different γ-CDs, due to the endothermic behavior which corresponded to the loss of water molecules in γ-CD’s surrounding and cavities. γ-CD, γ-CD-NHCH_2_CH_2_NH_2_, γ-CD-NHCH_2_CH_2_OH, and γ-CD-NH_2_ start to decompose at 313.00, 286.80, 302.00, and 306.00 °C, respectively. The chemical modification of γ-CD decreases the decomposition temperature of γ-CD [22].

Moreover, the TGA thermogram showed a complete degradation, with almost 80% weight loss for the native γ-CD, compared to a better stability of the modified γ-CD-NHCH_2_CH_2_NH_2_ and γ-CD-NHCH_2_CH_2_OH with around 75% weight loss. Meanwhile, γ-CD-NH_2_ showed a relatively low stability compared to other CDs, with 94.2% weight loss. The % of the residual mass at 400 °C was 23% for the native γ-CD, and 25%, 27%, and 15% for γ-CD-NHCH_2_CH_2_OH, γ-CD-NHCH_2_CH_2_NH_2,_ and γ-CD-NH_2,_ respectively.

### 2.2. Guest–Host Interaction between CUR and Modified γ-CDs by Absorption Spectra

**Figure 4:**
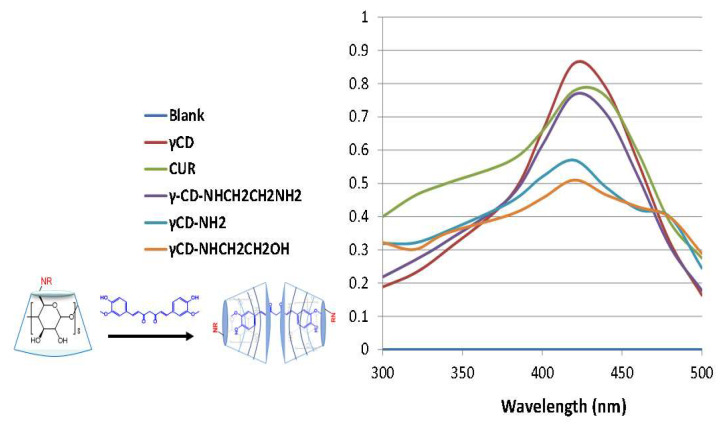
Absorption spectra of (0.04 mM) CUR, (0.34 mM), CUR-γ-CD-NH_2_, CUR-γ-CD-NHCH_2_CH_2_NH_2_, and CUR-γ-CD-NHCH_2_CH_2_OH.

The parent γ-CDs have no absorption in the range 300–500 nm, while CUR has a maximum absorption (λ_max_) at 420 nm; hence, the wavelength 420 nm was used to investigate the complex formation between modified γ-CDs and CUR. The effect of the increasing concentration of γ-CD, γ-CD-NHCH_2_CH_2_NH_2_, γ-CD-NHCH_2_CH_2_OH, and γ-CD-NH_2_ on the absorption spectrum of CUR was studied with a constant concentration of CUR. As a result of complexation, the chromophore, CUR, was transferred from the aqueous environment into the hydrophobic environment of the cyclodextrin cavity, causing a hypochromic or bathochromic shift, which are the commonly reported effects of the complex formation [23]. Figure 4 shows an increase in the CUR absorbance upon the addition of γ-CD. This may be attributed to the enhanced aqueous solubility of CUR upon transference of one or two aromatic rings (chromophores) of CUR from the aqueous medium to the non-polar cavity of γ-CD [23]. γ-CD showed a hyperchromic shift compared to the pure CUR, while the modified γ-CD showed a hypochromic shift compared to the pure CUR and the native γ-CD, with decreasing absorption values from γ-CD-NHCH_2_CH_2_NH_2_ to γ-CD-NH_2_ and γ-CD-NHCH_2_CH_2_OH. The variations in the absorption values may be attributed to the different complexation patterns between the three different types of the modified γ-CD-NH_2_. Moreover, these variations also indicate different forms of the modified γ-CD-NH_2_.

### 2.3. Determination of the Complexation Stoichiometry and Formation Constants

**Figure 5:**
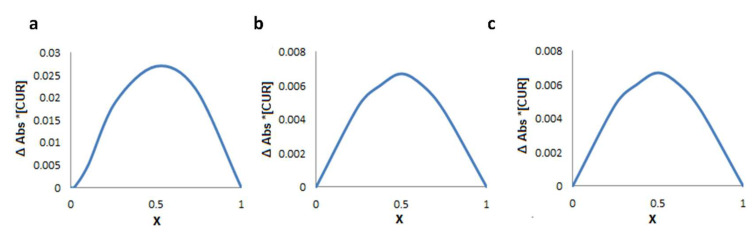
Job’s plots for (**a**) CUR-γCD-NH_2_, (**b**) CUR-γ-CD-CD-NHCH_2_CH_2_NH_2_, and (**c**) CUR-γ-CD-NHCH_2_CH_2_OH, where X is the mole fraction of CUR.

The continuous variation method (Job’s method) was employed to determine the complexation stoichiometry, as shown in Figure 5, where the CUR mole fraction (X) varied from 0.0 to 1.0 and was plotted against the absorbance difference (∆Abs) multiplied by the concentration of CUR. Job’s plot for the three different complexes showed a maximum peak at X = 0.5, which points to a 1:1 inclusion complex between CUR and different types of modified γ-CDs.

**Figure 6:**
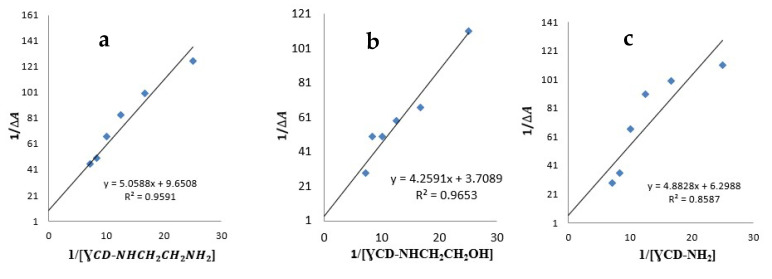
Benesi–Hildebrand plot of 1/∆*A* versus 1/[modified of γ-CDs] for CUR modified of γ-CDs inclusion complexes (**a**) γ-CD-NH_2_, (**b**) γ-CD-NHCH_2_CH_2_NH_2_, and (**c**) γ-CD-NHCH_2_CH_2_OH.

The binding constant (*K_f_*) values for the 1:1 stoichiometric ratio between modified γ-CDs and the CUR inclusion complex were determined using the Benesi–Hildebrand equation (Equation (1)), after plotting 1/ΔA versus 1/[modified γ-CDs] (Figure 6). For a 1:1 stoichiometry, the formation constant (*K_f_*) is equal to 8.98 ± 0.90 mM^−1^ for γ-CD-NHCH_2_CH_2_NH_2_, 5.85 ± 0.80 mM^−1^ for γ-CD-NHCH_2_CH_2_OH, and 6.70 ± 1.02 mM^−1^ for γ-CD-NH_2_.

The order of the increasing *K_f_* values were in good consistency with the UV absorption results, in which the highest hypochromic shift was observed with γ-CD-NHCH_2_CH_2_OH, which has the smallest *K_f_* value (5.85 mM^−1^). The same result was obtained with NHCH_2_CH_2_NH_2,_ which showed a low hypochromic shift with greater *K_f_* values, while γ-CD-NH_2_ was in between. The *K_f_* values and the UV absorption spectrum indicated different complexation patterns for different modified γ-CD, in which the amino groups, along with the ethylene arm, may sterically and electrostatically stabilize the CUR complex formation, as shown with NHCH_2_CH_2_NH_2_, compared to γ-CD-NHCH_2_CH_2_OH and γ-CD-NH_2_.

### 2.4. Cytotoxicity Assay

**Figure 7:**
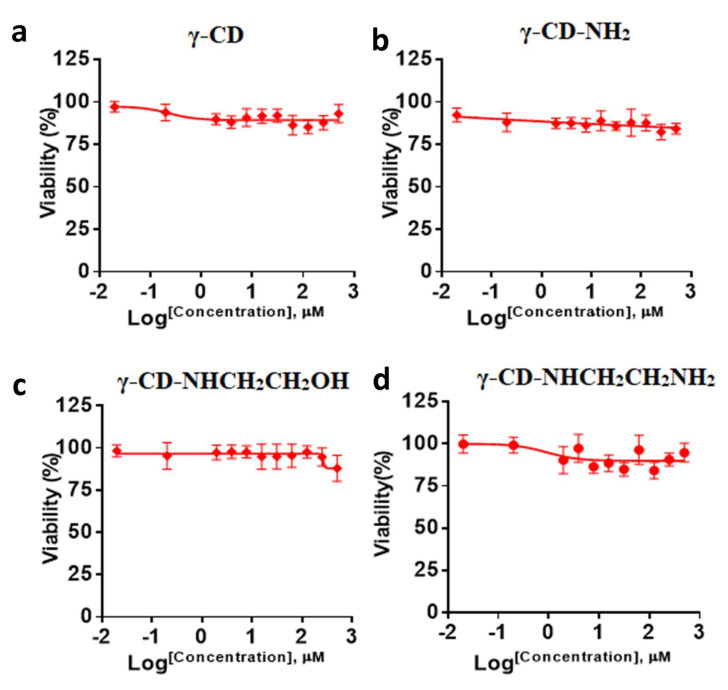
The dose–response curves of (**a**) γ-CD, (**b**) γ-CD-NH_2_, (**c**) γ-CD-NHCH_2_CH_2_OH, and (**d**) γ-CD-NHCH_2_CH_2_NH_2_ against HDF cells.

The cytotoxic effect of γ-CD, γ-CD-NHCH_2_CH_2_NH_2_, γ-CD-NH_2_, and γ-CD-NHCH_2_CH_2_OH was investigated against a normal human dermal fibroblast cell line (HDF) using an MTT assay. HDF cells were treated with different concentrations of γ-CD, γ-CD-NHCH_2_CH_2_NH_2_, γ-CD-NH_2_, and γ-CD-NHCH_2_CH_2_OH for 72 h. The results revealed that no significant toxicity of γ-CD, γ-CD-NHCH_2_CH_2_NH_2_, γ-CD-NH_2_, and γ-CD-NHCH_2_CH_2_OH was observed against HDF normal cells (Figure 7). These results confirmed the low toxicity of γ-CD-NHCH_2_CH_2_NH_2_, γ-CD-NH_2_, and γ-CD-NHCH_2_CH_2_OH and support the safe intended use as a drug carrier.

In conclusion, we have discussed the preparation and characterization of three amino-modified γ-CDs: mono-6-amino-6-deoxy-cyclodextrine (γ-CD-NH_2_); mono-6-deoxy-6-ethanolamine-γ-cyclodextrine (γ-CD-NHCH_2_CH_2_OH); and mono-6-deoxy-6-aminoethylamino)-γ-cyclodextrin (γ-CD-NHCH_2_CH_2_NH_2_). NMR and HR mass data confirmed that modified γ-CDs were successfully prepared. These modified γ-CDs also showed low toxicity against normal human dermal fibroblast cell lines (HDF), making them suitable as drug carriers. In addition, CUR (which is considered an extremely hydrophobic natural compound) was successfully encapsulated in these modified γ-CDs, proving that these modifications did not affect γ-CD’s ability to form guest–host complexes. The stoichiometry of the inclusion complexes was determined using Job’s plot and the Benesi–Hildebrand equation.

## 3. Materials and Methods

### 3.1. Chemicals

γ-cyclodextrin (γ-CD), curcumin, bis(4-hydroxy-3-methoxyphenyl)-1,6-diene-3,5-dione (CUR), and p-toluene-sulfonyl chloride (TsCl) were purchased from Sigma (St. Louis, MO, USA). Sodium azide, 1,1,2,2-tetrachloroethane, pyridine, N,N-dimethylformamide (DMF), phosphate buffer saline (PBS), 1-ethanol-2-amine ethanolamine, and ethylenediamine were purchased from TEDIA (Fairfield, OH, USA). Deuterated dimethylsulfoxide (d_6_-DMSO) (99.9% atom) was used for NMR analysis and was purchased from Aldrich (USA). Acetone, methanol, ethanol, acetonitrile, and 1-propanol were obtained from the Carbon Group (Carbon Group, Derbyshire, UK). All chemicals and solvents were of an analytical grade. All reagents and chemicals were used without further treatment.

### 3.2. Synthesis of Mono Amino Modified γ-CDs

The amino-modified γ-CDs, mono-6-amino-6-deoxy-cyclodextrine (γ-CD-NH_2_), mono-6-deoxy-6-ethanolamine-γ-cyclodextrine (γ-CD-NHCH_2_CH_2_OH), and mono-6-deoxy-6-aminoethylamino)-γ-cyclodextrin (γ-CD-NHCH_2_CH_2_NH_2_), were synthesized as illustrated in Scheme 1. Firstly, mono-6-(p-toluenesulfonyl)-6-deoxy-γ-cyclodextrin (6-OTs-γ-CD) was synthesized by tosylation of γ-CD with p-toluenesulfonyl chloride (TsCl) in freshly dried pyridine, according to the literature procedure [21], with some modifications. Adapting the procedure described in the literature using a Schlenk line, 5.00 g (3.86 mmol) of γ-CD with 0.70 g (3.67 mmol) of TsCl yielded 6-Ots-γCD as a white solid (1.40g, 25% yield). Pure 6-Ots-γ-CD was obtained after its recrystallization in methanol: water (1:1). The amino-modified γ-CD-NHCH_2_CH_2_NH_2_ and γ-CD-NHCH_2_CH_2_OH were synthesized by the nucleophilic substitution of 6-Ots-γ-CD according to the published procedure [22], using 6-Ots-γ-CD (0.24 mmol, 0.34g) with an excess of aliphatic amine 1-ethanol-2-amine (17.63 mmol, 1.06 mL), forming a white precipitate. Suction filtration followed by recrystallization in methanol: water (1:1) yielded (51%, 0.160 g). Mono-6-*O*-6-tosyl-γ-cyclodextrin (1.50 g, 1.17 mmol) was added to ethylenediamine (5.00 mL) under N_2_. The mixture was stirred at 60 °C for 12 h, and then cooled to room temperature. After cooling, the reaction mixture was poured into a large amount of ethanol to precipitate the product. The precipitate was collected by filtration, washed with ethanol, and dried under reduced pressure to give the product (1.21 g, 1.03 mmol) an 88% yield [19]. Mono-6-amino-deoxy-cyclodextrine (γ-CD-NH_2_) was prepared according to Tang et al. [21] with some modification. The reduction of mono-6-azido-6-deoxy-cyclodextrin (γ-CD-N_3_) was performed as illustrated in Scheme 1, where 1.00g (0.79 mmol) of 6-Ots-γCD was added to 1.00 g (15.78 mmol) of sodium azide. A white solid γ-CD-N_3_ was produced (0.5g, 54%). Recrystallization was performed in hot methanol. The white solid γ-CD-N_3_ was dried under a vacuum overnight at 60 °C. Mono-6-amino-6-deoxy-cyclodextrine (γCD-NH_2_) was synthesized by mixing 0.20 g (0.16 mmol) of γ-CD-N_3_, 0.046 g (0.18 mmol) of triphenylphosphine, and 0.32 mL DMF in a 50 mL one-neck round-bottomed flask. The product was precipitated using 3.3 mL of acetone, and then it was collected, washed again with acetone, and dried overnight to yield 0.161g (77%).

### 3.3. Nuclear Magnetic Spectroscopy (NMR)

All NMR spectra were obtained in deuterated solvents (d_6_-DMSO, D_2_O, and CD3OH (99.9% atom, Across Organics, Pittsburgh, PA, USA) using a Bruker Biospin AG Magnet system 500MHz/54mm instrument (Bruker Biospin, Fallanden, Switzerland) with a PA BBO 500S1 BBF-H-D-05 Z SP probe (Bruker Biospin, Fallanden, Switzerland), and the temperature was controlled using a variable temperature unit (VTU) and was held constant at 300 °K. Chemical shifts were measured in parts per million (ppm) and referenced to tetramethylsilane (TMS). The FID data were processed and analyzed using topspin Bruker software (Bruker Biospin GmbH, Billerica, MA, USA, version 4.1.3).

### 3.4. Mass Spectrometry

Positive or negative modes of high-resolution mass spectra (HR-MS) used the electrospray ion trap (ESI) technique by collision-induced dissociation on a Bruker APEX-4 (7 Tesla) instrument. The samples were infused using a syringe pump at a flow rate of 2 µL/min.

### 3.5. Thermo-Gravimetric Analysis (TGA)

The TGA analysis was performed by the Mettler–Toledo instrument (Mettler-Toledo, Columbus, OH, USA) over a temperature range of 25 °C to 400 °C with a heating rate of 10 °C/min. Alumina (aluminum oxide, ALU) crucibles were used to hold 1–2 mg of each sample.

### 3.6. Preparation of Encapsulation Complex

The complexes of CUR with different modified γ-CDs were performed via the solvent evaporation encapsulation technique. A 1:1 CUR to modified γ-CDs ratio (0.04 mmol, 16.00 mg) of CUR was dissolved in 1.0 mL methanol and 0.04 mmol of modified γ-CD (1.96, 2.00, and 2.00 mg for γ-CD-NHCH_2_CH_2_NH_2_, γ-CD-NHCH_2_CH_2_OH, and γ-CD-NH_2_, respectively), dissolved in 2.0 mL PBS buffer at pH 7.4. CUR solution was added dropwise into modified γ-CDs solutions then mixed by shaking at 40 °C for 24 h without evaporating the organic solvent completely. The uncomplexed drug was removed afterwards by centrifugation at 1500 rpm for 10 min [24,25].

### 3.7. Spectrophotometric Studies

The absorption spectrum, stoichiometry, and the formation constant of the inclusion complexes were determined by spectroscopic studies. The range of 300 to 500 nm using a Nanodrop 2000 UV–Vis spectrophotometer was utilized to obtain absorption spectra [26,27]. The continuous variation method (Job’s method) was employed to determine the complexation stoichiometry [23]. In this experiment, the samples were prepared by mixing various volumes of the two solutions (the same molar concentrations of the CUR and modified γ-CDs) to give the total concentration constant and the molar fraction of CUR, with X varying in the range from 0 to 1. The value of X gives the stoichiometry of inclusion complex (X = 0.5 for 1:1 ratio complex; X = 0.33 for 1:2 ratio complexes) [28]. The binding constant of the CUR-γ-CD inclusion complex was determined by the Benesi–Hildebrand equation [29]. The concentration of CUR was maintained at a constant (0.04 mM) throughout the experiment, whereas modified γ-CDs’ concentration varied from 1 to 3 molar ratios.

The binding constant (*K_f_*) for the 1:1 ratio between modified γ-CDs and the CUR inclusion complex was determined using the Benesi–Hildebrand (Equation (1)), using a plot of $\frac{1}{A-A_{o}}$ versus 1/[modified γ-CDs]

(1) $$\frac{1}{A-A_{o}}=\frac{1}{A^{\prime }-A_{o}}+\frac{1}{K_{f}\left(A^{\prime }-A_{o}\right)\left[\gamma -CD\right]}$$

where *A_o_* is the absorbance of the guest without modified γ-CDs, A is the absorbance with a particular concentration of modified γ-CDs, and *A*′ is the absorbance at the maximum concentration of modified γ-CDs.

The formation constant (*K_f_*) can be calculated using Equation (2).

(2) $$K_{f}=\frac{1}{slope\;\;\left(A^{\prime }-A_{o}\right)}$$

### 3.8. Cells Culture

Certified pure unloaded cyclodextrin compounds, γ-CD, γ-CD-NHCH_2_CH_2_NH_2_, γ-CD-NH_2_, and γ-CD-NHCH_2_CH_2_OH, were tested for their in vitro cytotoxic activities. The human dermal fibroblast cell line (HDF) was cultured in Dulbecco’s modified Eagle medium (DMEM) (Euroclone SpA, Pero, Italy). DMEM was supplemented with 10% (*v*/*v*) fetal bovine serum (FBS), 100 U/mL of penicillin, 100 mg/mL of streptomycin, and 2 mM of L-Glutamine, and was maintained in an incubator (Memmert, Büchenbach, Germany) at 37 °C under an atmosphere of 5% CO_2_ and 90% relative humidity. The cells were sub-cultivated approximately every 2 to 3 days using 0.05% (*w*/*v*) trypsin-EDTA.

### 3.9. Cell Viability Assay

HDF cells (5 × 10^3^ cells per well) were seeded in 96-well plates. After 24 h, the cells were treated with serial dilution of each of the γ-CD, γ-CD-NHCH_2_CH_2_NH_2_, γ-CD-NH_2_, and γ-CD-NHCH_2_CH_2_OH in the concentration range of 0.0 to 500 μM for 72 h. After 72 h of incubation at 37 °C, the treatment was removed from the wells, and was followed by adding 15 μL of 3-(4,5-Dimethyl-2-thiazolyl)-2,5-diphenyltetrazolium bromide (MTT) solution and 100.00 μL of the medium. After 3 h of incubation, the medium was removed, and then 50.00 μL of dimethyl sulphoxide (DMSO) was added to dissolve the formazan. The absorbance was measured at a wavelength of 570 nm using a Glomax microplate reader (Promega, Madison, WI, USA) [30].

HDF cells were treated with serial concentrations of γ-CD, γ-CD-NH_2_, γ-CD-NHCH_2_CH_2_OH, and γ-CD-NHCH_2_CH_2_NH_2_ for 72 h. The cell’s viability was determined by the MTT assay. Data mean ± SD (*n* = 3).

## Data Availability

Not applicable.

## Supplementary Materials

The following supporting information can be downloaded at, Figure S1: ^1^H NMR spectrum of γ-CD; Figure S2: ^13^C-NMR spectrum of γ-CD in d_6_-DMSO; Figure S3: ^13^C- dept 135-NMR spectrum of γ-CD in d_6_-DMSO; Figure S4: ^1^H-^15^N-HMBC spectrum of γ-CD-NHCH_2_CH_2_NH_2_ (in 80% d_6_-DMSO/D_2_O at 25 °C); Figure S5: ^1^H-NMR spectrum of γ-CD-NHCH_2_CH_2_OH in D2O at 25 °C; Figure S6: ^13^C-NMR spectrum of γ-CD-NHCH_2_CH_2_OH in d_6_-DMSO at 25 °C.

## Sample Availability

Samples of the compounds in this study are not available from the authors.
