# Supplementary material for: Synthesis of Mono-Amino Substituted γ-CD: Host–Guest Complexation and In Vitro Cytotoxicity Investigation
Source: Molecules. 2022 Mar 4;27(5):1683. doi: 10.3390/molecules27051683 (PMC8911948; doi:10.3390/molecules27051683)
Supplement: Supplementary file 1 [file molecules-27-01683-s001.zip › molecules-1554389-supplementary.pdf]

Supplementary Material

Synthesis of mono-amino substituted γ-CD: Host-guest complexation and *in vitro* cytotoxicity investigation

Figure S1: ^{1}H NMR spectrum of γ-CD

Figure S2: ^{13}C-NMR spectrum of γ-CD in d_6-DMSO

Figure S3: ^{13}C- dept 135-NMR spectrum of γ-CD in d_6-DMSO

Figure S4: ^{1}H-^{15}N-HMBC spectrum of γ-CD-$NHCH_2CH_2NH_2$ (in 80% d_6-DMSO/D_2O at 25 °C)

Figure S5: ^{1}H-NMR spectrum of γ-CD-$NHCH_2CH_2OH$ in D_2O at 25 °C

Figure S6: ^{13}C-NMR spectrum of γ-CD-$NHCH_2CH_2OH$ in d_6-DMSO at 25 °C

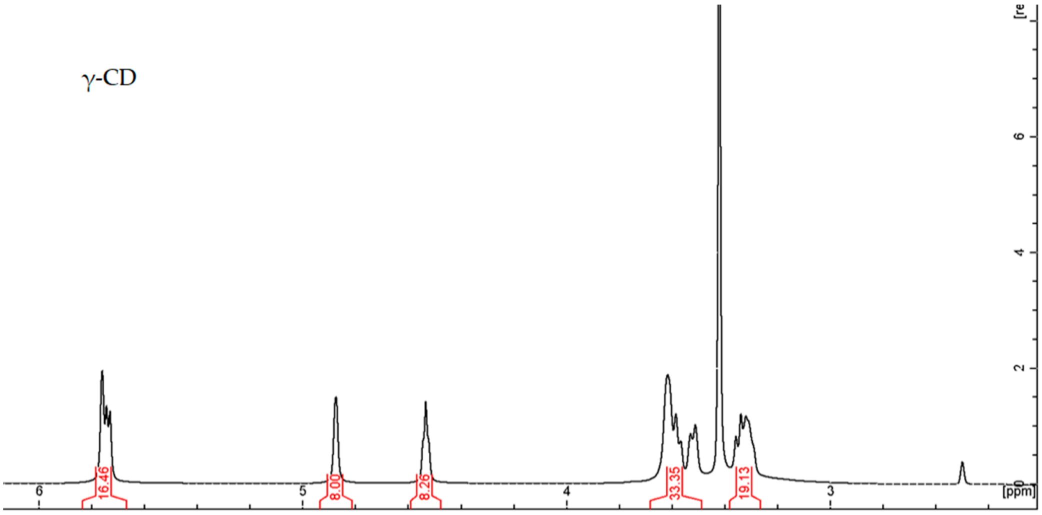

Figure S1: ^{1}H-NMR spectrum of γ-CD in d_6-DMSO

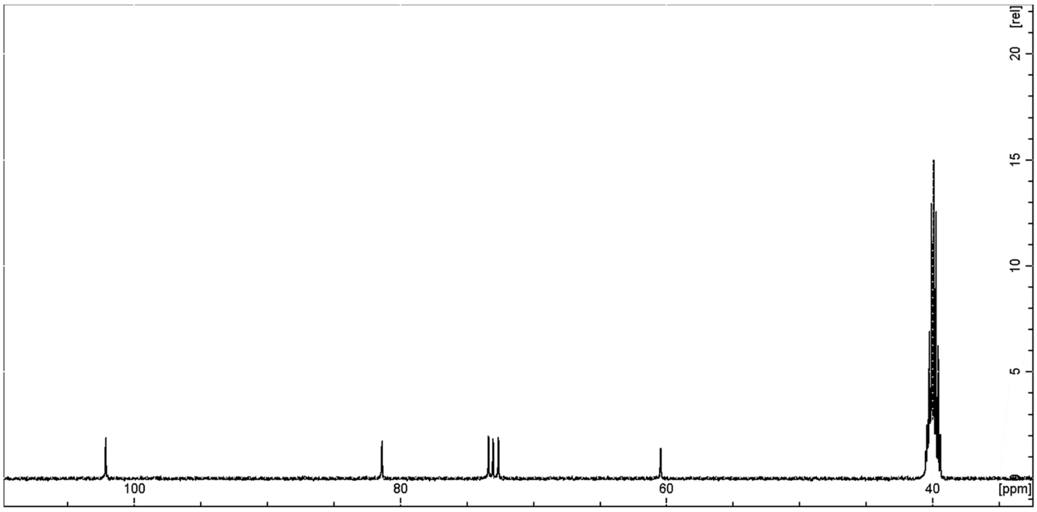

Figure S2: ^{13}C-NMR spectrum of γ-CD in d_6-DMSO

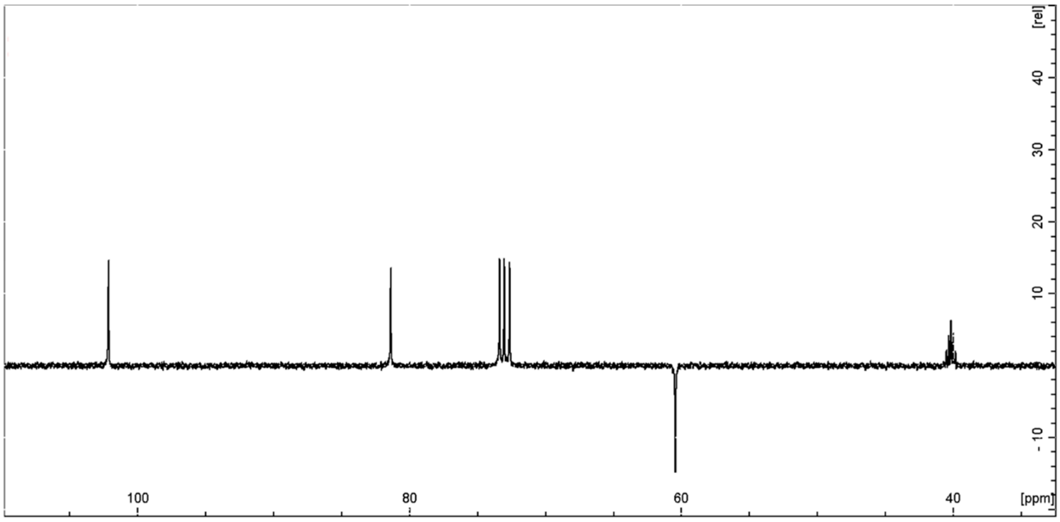

Figure S3: ^{13}C- dept 135-NMR spectrum of γ-CD in d_6-DMSO

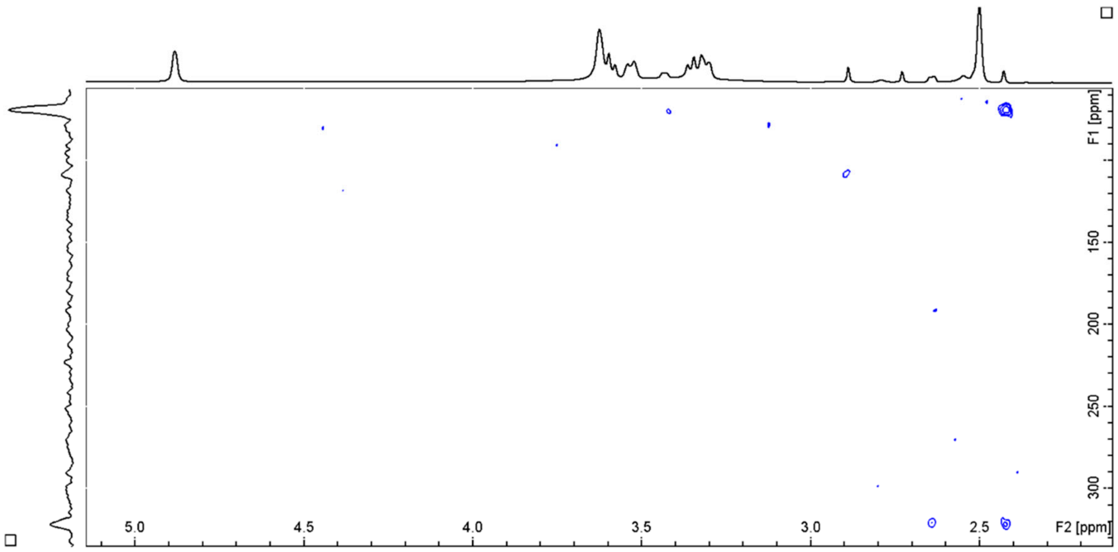

Figure S4: ^{1}H-^{15}N-HMBC spectrum of γ-CD-NHCH$_2$CH$_2$NH$_2$ (in 80% d_6-DMSO/D_2O at 25 °C)

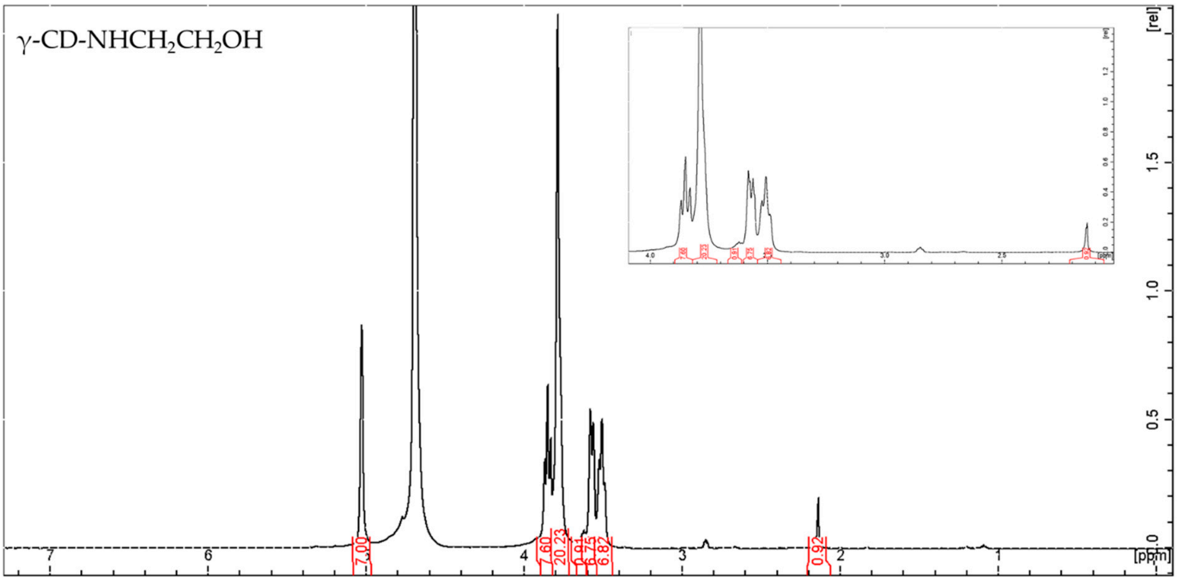

Figure S5: ^{1}H-NMR spectrum of γ-CD-NHCH$_2$CH$_2$OH in D_2O at 25 °C

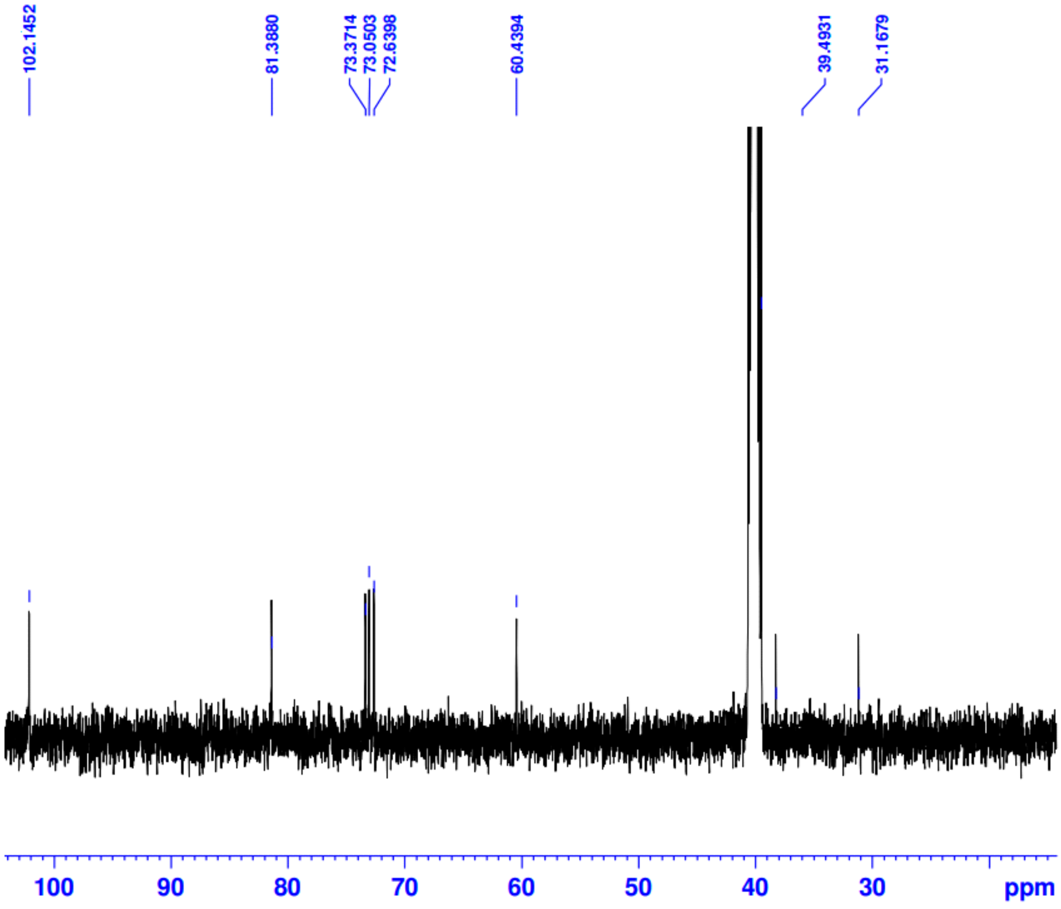

Figure S6: ^{13}C-NMR spectrum of γ-CD-NHCH$_2$CH$_2$OH in d_6-DMSO at 25 °C
